# More than Just Protein Degradation: The Regulatory Roles and Moonlighting Functions of Extracellular Proteases Produced by Fungi Pathogenic for Humans

**DOI:** 10.3390/jof9010121

**Published:** 2023-01-15

**Authors:** Dorota Satala, Grazyna Bras, Andrzej Kozik, Maria Rapala-Kozik, Justyna Karkowska-Kuleta

**Affiliations:** 1 Department of Comparative Biochemistry and Bioanalytics, Faculty of Biochemistry, Biophysics and Biotechnology, Jagiellonian University, 30-387 Kraków, Poland; 2 Department of Analytical Biochemistry, Faculty of Biochemistry, Biophysics and Biotechnology, Jagiellonian University, 30-387 Kraków, Poland

**Keywords:** *Candida*, *Aspergillus*, dermatophytes, Sap, yapsins, proteinases, allergens

## Abstract

Extracellular proteases belong to the main virulence factors of pathogenic fungi. Their proteolytic activities plays a crucial role in the acquisition of nutrients from the external environment, destroying host barriers and defenses, and disrupting homeostasis in the human body, e.g., by affecting the functions of plasma proteolytic cascades, and playing sophisticated regulatory roles in various processes. Interestingly, some proteases belong to the group of moonlighting proteins, i.e., they have additional functions that contribute to successful host colonization and infection development, but they are not directly related to proteolysis. In this review, we describe examples of such multitasking of extracellular proteases that have been reported for medically important pathogenic fungi of the *Candida*, *Aspergillus*, *Penicillium*, *Cryptococcus*, *Rhizopus*, and *Pneumocystis* genera, as well as dermatophytes and selected endemic species. Additional functions of proteinases include supporting binding to host proteins, and adhesion to host cells. They also mediate self-aggregation and biofilm formation. In addition, fungal proteases affect the host immune cells and allergenicity, understood as the ability to stimulate a non-standard immune response. Finally, they play a role in the proper maintenance of cellular homeostasis. Knowledge about the multifunctionality of proteases, in addition to their canonical roles, greatly contributes to an understanding of the mechanisms of fungal pathogenicity.

## 1. Fungal Proteases Are Important Virulence Factors

Among the wide range of virulence factors found in pathogenic fungi, hydrolytic enzymes are a key component contributing to the invasiveness of these microorganisms. Phospholipases, lipases, proteases, hemolysins, phosphatases, ureases, and other hydrolases are an effective fungal arsenal for successful penetration, survival, and dissemination within the host organism [1,2,3]. Extracellular proteases are primarily important for the acquisition of nutrients from the environment, and they are necessary for the proper growth and development of fungal cells. Furthermore, they are also strongly involved in host tissue damage and overcoming host defense barriers, including the epithelium, basement membrane, interstitial matrix, and endothelial cell layer, facilitating fungal spread and progression of the infection. Proteolytic activity also allows for the effective evasion of the host immune system through the degradation of host defense proteins and peptides, and the dysregulation of plasma proteolytic cascades [4]. Thus, these enzymes can be considered as actually multitasking. However, these are functionalities that directly and naturally result from the degradative potential of fungal proteases, which, however, depends on the substrate specificity of individual enzymes. For some proteases, it may be broad, making their action multifaceted, while other proteolytic enzymes are more specifically targeted at selected substrates and may participate in a refined manner in the regulatory actions of various processes [5].

**Figure 1:**
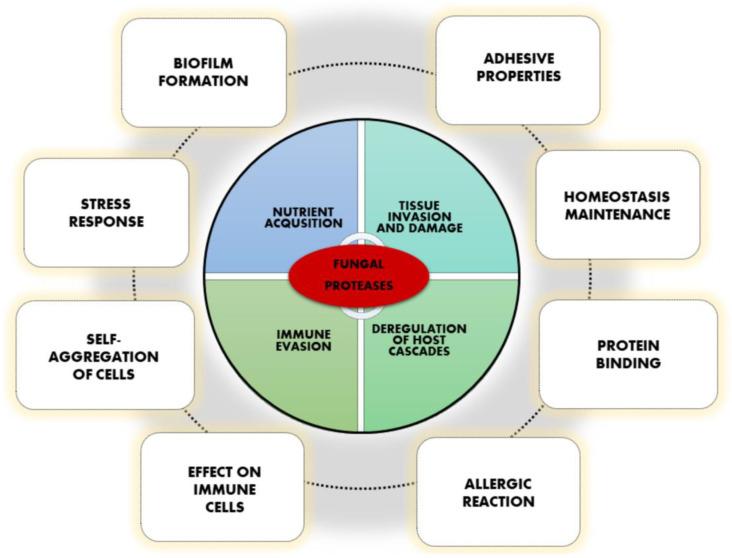
Various functions of fungal proteases important in virulence.

Recently, several reports have revealed new additional functions of fungal proteases that are important during infection, albeit not directly related to their proteolytic activity and destructive abilities (Figure 1) [4]. Although numerous different proteins could be designated as being bifunctional or multifunctional, a quite specific group called moonlighting proteins stands out among them and could be distinguished on the basis of performing an additional function, essentially distinct from the primary, evolutionary conserved activity, and often in a diverse cellular localization [6,7]. Moonlighting proteins are not pleiotropic proteins, splice variants, or effects of gene fusion or products of proteolytic fragmentation, and they often do not occur repeatedly between taxonomically related organisms [6,8]. Unraveling the novel functions of fungal proteases may place these enzymes not only within a diverse group of multifunctional proteins, but may even assign the moonlighting protein attributes to selected representatives, primarily on the basis of their proteolytic-independent roles. A similar phenomenon has also been demonstrated for bacterial proteases, for which, in addition to proteolysis, participation in adhesion, protein folding, or cell signaling are frequently reported [9].

The canonical catalytic activity of proteases is limited to a relatively small area of the molecule; other remaining surfaces may be involved in regulatory functions, extracellular secretion, and protease deposition in a specific cellular location, as well as in interactions with the hydrolyzed substrate, and they might be also convenient for creating alternative functionalities [10]. Presumably, the selective pressure on the establishment of novel beneficial functions by evolutionarily old proteins and the tendency to limit the energy costs by cells, related to the use of existing molecules for divergent purposes, might also contribute to the emergence of new, nonclassical functions of fungal proteases [10,11]. The involvement of protein multifunctionality in the virulence process may be particularly useful for microbial pathogens, which can thus achieve even more benefits during host invasion by using not only the available arsenal of classic virulence factors with their canonical activities, but also by exploiting the additional potential of produced proteins. In this review, we focus mainly on the non-canonical functions of fungal proteases, distinct from their evident proteolytic activities, and that are important in the pathogeneses of fungal infections, which further determine the classification of certain proteases as moonlighting proteins. In addition, we also refer to the proteolytic activity of fungal enzymes that are not directly related to the degradation of different substrates, but that are based on the more subtle modulation of various mechanisms contributing to the enhancement of fungal virulence.

Currently, several hundred species of fungi are considered as being important infectious agents that endanger the human population, being responsible for various types of infections, including burdensome superficial mycoses and difficult-to-treat invasive fungal infections that are associated with high mortality rates [12,13]. Among the medically important fungi are the *Candida* species, including the most widely distributed *C. albicans*, as well as the emerging non-*albicans Candida* yeasts; furthermore, the ubiquitous fungi *Aspergillus fumigatus*, *Cryptococcus neoformans*, *Pneumocystis jiroveci*, and dermatophytes; and notable endemic fungal species from the genera *Paracoccidioides, Blastomyces*, Histoplasma, *Coccidioides*, and *Sporothrix* [14]. These microorganisms produce a wide variety of proteases belonging to different classes, distinguished on the basis of the catalytic mechanism (Table 1). For some particular fungal proteolytic enzymes, multifunctionality has been frequently demonstrated, as well as its significant contribution to fungal virulence. The best documented examples of such additional functionalities for extracellular proteases produced by selected fungal species are discussed below.

## 2. Involvement in Binding, Adhesion, Self-Aggregation, and Biofilm Formation

The ability of fungi to adhere to the surface of host cells and tissues, as well as to bind to host proteins and to produce biofilms on various surfaces, is crucial for the development of fungal infection. Fungal pathogens have exploited a number of different mechanisms that facilitate adhesion, based primarily on the production of specialized adhesive proteins exposed on the surfaces of fungal cells—mainly typical adhesins belonging to glycosylphosphatidylinositol (GPI)-anchored cell wall proteins, and also the involvement of other cell wall components, including polysaccharides, as well as general cell surface hydrophobicity, and electrostatic and van der Waals forces [15]. Furthermore, the cooperation of surface moonlighting proteins, many of which are cytoplasmic enzymes by origin, such as enolase, triosephosphate isomerase, or aldolase, also contributes to enhanced adhesion, as has been repeatedly demonstrated for different fungi [7,16]. In addition, it has been shown that fungal proteases may also reveal extraordinary functions that are related to adhesion.

To date, at least two proposed explanations are considered on how fungal proteases can support adhesion to host cells. First, that secreted proteases have the ability to proteolytically modify the surfaces of host cells or fungal cells to reveal proteins that are more suitable ligands for fungal binding or more efficient pathogen adhesins, respectively. Otherwise, fungal cell surface proteases themselves may serve as ligands that are involved in the binding of host cells or proteins, independently of their proteolytic activities [2,17]. The latter hypothesis allows for the assignment of moonlighting functions to particular proteases, while the former is based on the canonical functions of these proteins, which is proteolytic activity, although directed at the modulation of the properties of fungal cells related to their virulence, and interactions with the host and the environment. Furthermore, the overlapping mechanisms of the participation of proteases in the adhesion phenomenon cannot be excluded either.

Extensively studied examples of multifunctional proteases are secreted aspartyl proteases (Saps) produced by *Candida* species, which are involved in numerous roles related to the regulation of cell adhesion and biofilm formation, also in complex environments. The results presented for *C. albicans* have indicated that fungal biofilm formation was positively associated with the secretion of proteases [18], and individual proteases from the Sap family were often involved in adhesion and invasion on epithelial cells [19,20]. *C. albicans* Sap9 and Sap10, which are GPI-anchored glycosylated proteins located in the cell membrane and/or the cell wall, unlike other members of the Sap family, were indicated to play an important role in the maintenance of fungal cell wall integrity, since mutants lacking these proteinases exhibited impaired growth in the presence of substances that affect the cell wall, including hygromycin B, calcofluor, Congo red, or itraconazole, and they were characterized by the increased level of cell wall chitin [21]. Additionally, these mutants demonstrated altered binding and invasion of epithelial cells; since *SAP9*-deficient cells showed increased adhesion, *SAP10*-deficient cells presented a lower level of adhesion compared to the wild-type strain, while the mutant lacking both proteases also had reduced adhesion; however, both single mutants presented a reduced capacity to invade and to damage reconstituted human epithelium in the model of oral infection [21]. Although Albrecht et al. [21] did not exclude the action of Sap10 as a typical adhesin, the influence of both proteinases on the adhesion of fungal cells was attributed mainly to their enzymatic activity and the proteolytic modulation of fungal surface proteins, which was particularly significant in the light of further reports on the abilities of Sap9 and Sap10 to hydrolyze a wide variety of covalently linked GPI-modified surface proteins, including chitinase Cht2, transglucosidase Pga4, or Als2 adhesin from the agglutinin-like sequence (Als) family [22]. Therefore, the results from the work of Schild et al. [22], describing the influences of Sap9 and Sap10 on the maintenance of chitinase activity, corroborated previous findings describing the increase in the amount of chitin in the cell wall in mutants lacking both proteases [21]. Furthermore, a *C. albicans* mutant strain lacking *SAP9* displayed a reduction in yeast-to-hyphae transformation under serum hypha-induced conditions, and a noticeable downregulation of the transcription factor Efg1, essential in inducing the process of morphological transition [23,24]. As a consequence, this resulted in a significant decrease in the endocytosis of the *sap9*Δ/Δ mutant strain by host oral epithelial cells [24]. Sap9 was also indicated as one of the factors that are involved in protein–receptor interactions between fungal cells, fungal biofilm formation, and in the interactions of *C. albicans* with other microorganisms in mixed species biofilms [25]. Mono-species biofilms formed by the *C. albicans* mutant strain deprived of *SAP9* were characterized as being more compacted, and with a larger number of condensed hyphae, when compared to a wild type strain, for which biofilms were more spatially developed. Similarly, such an observation was also reported for mixed biofilms formed with the bacteria of *Streptococcus* species; again, the biofilm architecture for the mutant strain was somewhat different, with more bacterial cells being accumulated on the saliva-coated cover slips, than was observed for the mixed biofilm formed by the wild type *C. albicans* strain and bacteria. Thus, the obtained results implied that Sap9 might be advantageous in facilitating *C.* albicans competition with streptococci for salivary pellicle colonization [25].

For other members of the *C. albicans* Sap family, it has been shown that Sap7 facilitated fungal adhesion to the monolayers of colon adenocarcinoma-derived cells in the initial stages of invasion [26]. Additionally, Sap1 was demonstrated to be involved in the colonization of skin in a cutaneous mouse model, and the adhesion step was independent of Sap1 proteolytic activity, contrary to the stage of cavitation on the skin surface, while both mechanisms were required for skin invasion [27,28]. Furthermore, for *C. albicans*, Sap4-Sap6 proteinase adhesive properties were repeatedly reported, as these proteinases are equipped with amino acid sequences RGD/KGD that are identified as integrin-binding fragments. Sap4 has one RGD motif and Sap5 contains the RGDKGD sequence, while Sap6 has two sequential RGD motifs, all of which are located at the surface-exposed tip of the arm 1 loop situated close to the cavity with the enzyme active site [29]. These motifs were found as being responsible for the binding of Sap4-Sap6 to integrins localized on the surfaces of oral epithelial cells. After this interaction, proteinases were internalized to endosome and lysosome, and then they permeabilized the lysosomal membrane, leading to the apoptosis of host cells [29]. Additionally, Kumar et al. [17] reported that in a murine model of oral candidiasis, the *C. albicans* mutant strain overexpressing *SAP6* formed thicker fungal plaques than the mutant strains lacking Sap6 enzyme. Moreover, Sap6, independently of its proteolytic activity, enabled the aggregation of *C. albicans* cells—the effect that was assigned to RGDRGD sequence binding to germinated fungal cells [17]. Furthermore, for Sap6, the ability to bind zinc ions was also revealed, as well as the presence of four amyloid-forming regions, which, in the presence of zinc, were additionally responsible for fungal cell aggregation [30]. Hence, Sap6 can be considered as the truly multifunctional, and even moonlighting protein responsible for the assembly of cells in the biofilm, regulating adhesion and the uptake of zinc [30].

**Figure 2:**
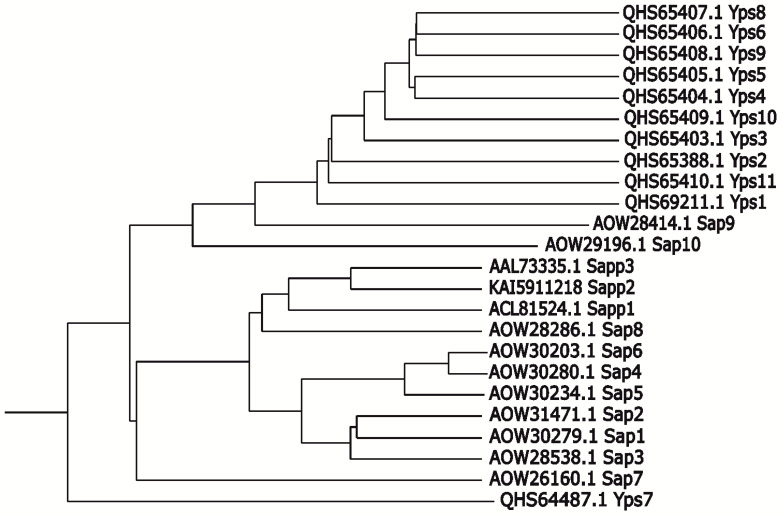
Dendrogram presenting the relationship of *Candida* spp. extracellular proteases. Prepared using the T-REX tool (http://www.trex.uqam.ca/) [37].

For *C. parapsilosis*, with the use of mutant strains with the expression of only a single aspartic proteinase—Sapp1, Sapp2, or Sapp3, the participation of Sapp1 and Sapp2, but not Sapp3, in the binding of *C. parapsilosis* to the TR146 oral epithelial cell line was demonstrated; however, the mechanisms of these interactions require further elucidation [31]. Of these secreted enzymes, Sapp1 was indicated as being temporarily embedded within the *C. parapsilosis* cell wall, and capable of operating in the immediate vicinity of the fungal cells; therefore, its functionality may be similar to those described for Sap9 and Sap10 of *C. albicans*; nonetheless, this issue requires further research [32]. For *C. glabrata*, a family of eleven GPI-linked aspartyl proteases, referred to as yapsins (CgYps) were identified, and their similarity to *C. albicans* Sap9 and Sap10 proteases was also proposed (Figure 2) [33,34,35]. On the basis of analyses performed using *C. glabrata* mutant strains with the deletion of genes encoding representatives of CgYps, especially *CgYPS1* and *CgYPS7*, it has been shown that CgYps play a key role in the colonization, dissemination, and long-term maintenance of *C. glabrata* in the kidney, liver, and spleen, in a mouse model of systemic infection [33,36]. Furthermore, CgYps participated in the colonization of brain tissues [36]. Additionally, the *Cgyps1*-*11*Δ mutant was shown to be impaired in biofilm formation on abiotic surfaces, while *CgYPS1* expression fully complemented this defect [36]. Thus, it seems that this proteinase plays an indirect role in biofilm formation by acting as a modulator of β-glucan exposition on the yeast cell wall [36].

For fungal species other than *Candida*, there have also been a few reports on the involvement of proteases in adhesion, but their mechanisms of interaction were not always identified, and it was not determined whether they are dependent on or independent of proteolytic activity. For *Microsporum canis,* a dermatophyte capable of infecting cats, dogs, and humans, the involvement of the secreted subtilisin Sub3 in fungal adhesion to the feline epidermis was demonstrated, although it was shown not to be crucial for invasion on host cells [38,39]. Nevertheless, the exact mechanisms of Sub3 adhesion have not yet been identified, and it is not clear whether it is based on the ability to perform the proteolytic modeling of other surface molecules, or whether Sub3 itself acts as a ligand for binding [40]. With the use of immunoproteomics, *Aspergillus fumigatus* alkaline protease 1 (Alp1/Asp f 13/oryzin) was identified as one of the fibrinogen-binding proteins, involved in the interactions of fungi with the human extracellular matrix, and thus promoting fungal growth, the adherence of fungal conidia, and infection development [41]. For *Cryptococcus neoformans*—an encapsulated fungus responsible for pneumonia and meningitis in immunocompromised individuals—the activity of serine protease, as well as secreted metalloprotease Mpr1, was demonstrated as being pivotal for interactions with microvascular endothelial cells and the disruption of the blood–brain barrier, facilitating subsequent fungal traversal to the central nervous system [42,43]. Moreover, Mjokane et al. [44,45] speculated recently that the activity of uncharacterized serine protease from *C. neoformans* may activate the SARS-CoV-2 spike (S) protein, with consequent membrane fusion and the endocytosis of virial particles by host cells, thus facilitating the development of the coronavirus disease COVID-19 in patients affected with concomitant fungal infection [44,45].

## 3. Effect on Cells of the Host Immune System

**Figure 3:**
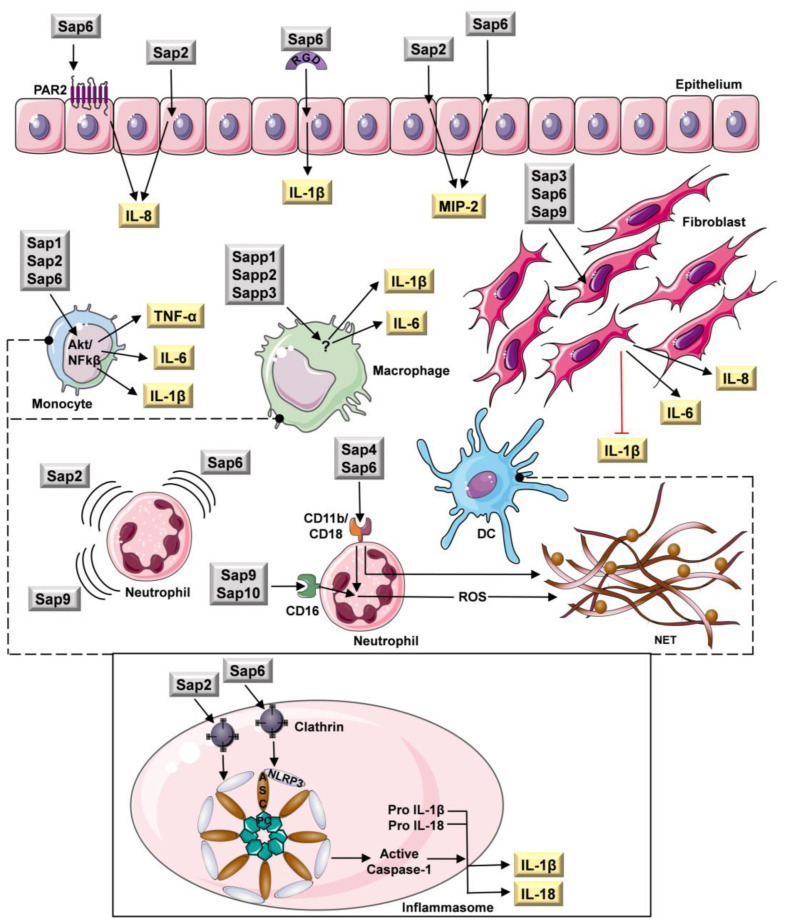
Multitasking of *Candida* spp. extracellular proteases in relation to the host immune cells. The figure was partly generated using Servier Medical Art, provided by Servier, licensed under a Creative Commons Attribution 3.0 unported license. ASC—apoptosis-associated speck-like protein containing a CARD, DC—dendritic cells, IL—interleukin, NET—neutrophil extracellular traps, NLRP3—intracellular NOD-like receptor 3, PAR2—proteinase-activated receptor 2, PC—pro-caspase-1, ROS—reactive oxygen species, Sap—secreted aspartyl proteinases, TNF-α—tumor necrosis factor α, MIP-2—macrophage inflammatory protein 2.

During the development of infection, pathogenic fungi are constantly exposed to varied activities of the host immune system, including the action of numerous molecules involved in the immune response and various types of host defense cells, aimed at destroying the microbial invaders. The human immunity directed at fungi comprises innate immunity and adaptive immunity; the former includes the complex antimicrobial activities of phagocytic cells—neutrophils, mononuclear leukocytes (monocytes and macrophages), and dendritic cells (DC). There is also the involvement of natural killer cells, γδ T cells, epithelium and endothelium, the action of cellular pattern recognition receptors, and the antigen presentation and production of cytokines and chemokines (reviewed in detail in [46]). Several mechanisms used by fungi to evade the host immune system are directly related to the proteolytic degradation of host defense proteins, i.e., immunoglobulins, lactoferrin, lactoperoxidase, components, and the regulators of the complement system and antifungal peptides, resulting in their inactivation [4]. However, it has been demonstrated that fungal proteases may also be involved in more refined interactions with the host immune system, affecting host cells and modulating their functionalities, as well as modifying the effectiveness of cytokines, based not only on proteolysis, but also independently of enzymatic activity. For instance, fungal proteases are able to regulate the host inflammatory cytokine response, not only via proteolytic activation of the interleukin 1β (IL-1β) precursor [47] or the degradation of various cytokines [48], but also by stimulating different cells to secrete these molecules, or by inhibiting their production (Figure 3). For example, fungal proteases may activate dedicated receptors present at the surfaces of different types of host cells—protease-activated receptors (PARs)—triggering the intracellular response through nuclear factor-κB (NF-κB), and the production of a variety of cytokines and chemokines that are capable of further stimulation of dendritic cells [49,50,51]. PARs are transmembrane proteins belonging to the G-protein-coupled receptor (GPCR) family, the activation of which consists of the targeted proteolysis of the extracellular N-terminus, followed by the exposition of an intramolecular tethered ligand and further intracellular signal transduction [52].

However, it was also demonstrated that *C. albicans* Sap1-Sap3 and Sap6, independent of their proteolytic activity, pH optima, and PAR activation, possessed the ability to induce human monocytes to the secretion of the proinflammatory cytokines IL-1β and TNF-α, via Akt/NF-κB activation, and that they were also capable of inducing Ca^2+^ influx. In the same studies, it was also shown that similarly, Sap1, Sap2, and Sap6 stimulated these host cells toward the production of interleukin 6 (IL-6) [53]. IL-1β was also secreted by oral epithelial cells after contact with Sap6, independent of its enzymatic activity. The interaction of host cells with the RGD integrin-binding domain of the Sap6 molecule was considered as being essential to initiate this process [54,55]. In the case of fibroblasts, Sap3, Sap6, and Sap9 stimulated the secretion of IL-6, but restrained the production of IL-1β; however, the mechanism of interactions was not indicated [56]. For *C. parapsilosis*, there is also the indirect evidence of the stimulation of cytokine production in response to secreted proteinases. Singh et al. [31] demonstrated that macrophages stimulated with mutants deprived of *SAPP1*-*SAPP3* produced significantly less IL-1β and IL-6, and moderately, although not significantly, less IL-8 than after stimulation with the wild-type strain. Fungal proteinases can also stimulate host cells to the production of chemokines, which induce neutrophil influx to the site of invasion. Gabrielli et al. [57] demonstrated that *C. albicans* Sap2 and Sap6 induced the secretion of IL-8 and macrophage inflammatory protein 2 (MIP-2) by vaginal epithelial cells. This phenomenon was partially reduced in the presence of pepstatin A, which is an inhibitor of aspartic proteases. This suggests an at least partial contribution of the proteolytic activities of Sap2 and Sap6 to this stimulation [57]. Moreover, Kumar et al. [55] showed that Sap6 also stimulated the oral epithelial cells to IL-8 production, albeit probably via a mechanism dependent on its protease activity and p38/c-Fos signaling, which was initiated by the proteolytic activation of PAR-2 localized on the cell surface. The enhanced secretion of IL-8 was also observed after the contact of fibroblast with Sap3, Sap6, and Sap9 [56]. In addition, there are several reports of fungal proteinases possessing chemoattractant properties, similar to chemokines [58]. Hornbach et al. [59] demonstrated that Sap9 directly stimulated neutrophils to chemotaxis toward *C. albicans* [59].

In a murine vaginitis model, the same activity was demonstrated also for Sap2, when neutrophils were found to migrate to the infection site in response to this proteinase [60]. Additionally, Gabrielli et al. [57] demonstrated that Sap2 and Sap6 might also act as direct chemoattractants in vitro, and that their enzymatic activities were not necessary for the Sap-induced migration of neutrophils, which allows them to be referred to as moonlighting proteins.

Inflammasome NLRP3 is proinflammatory multiprotein complex consisting of intracellular NOD-like receptor 3 (NLRP3), and apoptosis-associated speck-like protein containing a CARD (ASC) and pro-caspase-1 (PC). After activation, caspase-1 converts the precursors of IL-1β and interleukin 18 (IL-18) to the active forms. The complex is activated by diverse stimuli, including ionic flux, mitochondrial dysfunction, the generation of reactive oxygen species (ROS), and lysosomal damage [61]. Pietrella et al. [62] demonstrated that the NLRP3 inflammasome may be directly activated by Sap2 and Sap6. Both enzymes activated NLRP3 and caspase-1, after internalization mediated by clathrin, with the consequent secretion of maturated IL-1β and IL-18. This phenomenon was observed in monocytes, macrophages, and dendritic cells, regardless of pepstatin A presence, indicating that it proceeded independently of the proteolytic activities of the fungal enzymes [62]. A non-canonical inflammasome activation pathway through type I interferon production and the activation of caspase-11 that activates caspase-1 was also reported for Sap2 and Sap6 in murine macrophage cell lines. This indirect stimulation also required the internalization of proteinases [63]. As reported, macrophages expressed caspase-11 in response to both Sap2 and Sap6, and the response was more extended for Sap2. Importantly, the activation of caspase-11 did not affect Sap-induced NLRP3 expression, but it also potentiated caspase-1 activation, and as a result, the secretion of proinflammatory cytokines [63].

Hornbach et al. [59] reported that the deletion of the *SAP9* gene reduces the *C. albicans*-mediated apoptosis of human polymorphonuclear neutrophils (PMNs) via the inhibition of ROS release, while the process of programmed cell death is fundamental in the regulation of neutrophil homeostasis and balancing infection [59]. It has also been shown that the contact of neutrophils with proteinases Sap4, Sap6, Sap9, and Sap10 elicited an effective cell response to microbial stimuli and neutrophil extracellular traps (NETs) generation, which are fibrils composed of decondensed chromatin and granular proteins possessing strong antimicrobial activity. NET production was stimulated independently of Sap proteolytic activity, and at least partially through a ROS-dependent mechanism [64]. Neutrophil activation was proceeded by the CD11b (macrophage-1 antigen, Mac-1) receptor, and additionally, the CD11a (lymphocyte function-associated antigen 1, LFA-1), CD18, CD14, CD16, and TLR2 receptors. The collaboration of different types of neutrophil receptors in response to Saps resulted in signal transduction via the activation of tyrosine kinases Src and Syk, followed by the activation of pathways involving phosphoinositide 3-kinase (PI3K) and extracellular signal-regulated kinases ERK1/2 [64].

The main role of the family of CgYps has been repeatedly attributed to their functions in maintaining the proper structure and composition of the fungal cell wall [33,36,65,66,67]. The *CgYPS*-deficient mutant *CgYPS1*-*11*Δ under the conditions of the logarithmic growth phase presented significantly lower levels of β-glucan and mannan in the yeast cell wall, with a simultaneous increase in the chitin content [36]. Furthermore, the expression under its own promoter and ectopic expression confirmed that CgYps1 is required to maintain an appropriate β-glucan content [36,65], while CgYps1 with CgYps7 play a key role in maintaining levels of chitin and mannan [36]. Since components of the yeast cell wall are known to regulate the innate immune response, and given that *C. glabrata* exhibits a remarkable ability to survive and proliferate inside macrophages [68], it could be speculated that the preservation of an appropriate composition of the cell wall by CgYps1 could be aimed at attracting to the site of infection of the immune cells, capable of recognizing *C. glabrata* β-glucan via the Dectin-1 receptor [69,70]. Contrary, the absence of Sap9 and Sap10 proteases in the cell wall of *C. albicans* did not affect the surface exposition of β-1,3-glucan, and thus, interactions with macrophages [22]. The consequence of the internalization of *C. glabrata* cells inside host immune cells was the up-regulation of pathogen CgYPS-encoding genes, including *CgYPS2*, *CgYPS4*, *CgYPS5*, and *CgYPS8-11* for fungi residing in macrophages; and *CgYPS1, CgYPS2*, *CgYPS4-6*, and *CgYPS8-11* for fungal internalization by neutrophils [33,71]. Site-directed mutagenesis identified the putative catalytic aspartate residue of CgYps1 at position 91 involved in cell wall metabolism as being crucial for the intracellular survival and proliferation of yeasts [36]. Interestingly, in this context, Sapp1 and Sapp2, but not Sapp3, inhibit phagosome–lysosome fusion, and may promote the intracellular survival of *C. parapsilosis* in human macrophages [31].

In addition, the important role of *CgYPS* in suppressing the host innate immune response has been demonstrated. The infection of human THP-1 macrophages with the *C. glabrata Cgyps1*-*11*Δ mutant strain caused an increased production of the pro-inflammatory cytokine IL-1β, dependent on the activation of spleen tyrosine kinase (Syk) signaling, compared to the wild-type strain [36]. Therefore, the inability of the *Cgyps1*-*11*Δ mutant to survive and proliferate within mouse and human macrophages might be correlated with the activation of the NLRP3 inflammasome [36,72]. Furthermore, recent studies have shown that *CgYPS* plays an important role in the regulation of glucose homeostasis in fungal cells [67], while maintaining an adequate level of glucose metabolism is crucial for both fungi and host immune cells during *Candida* invasion, supporting host survival in life-threatening systemic candidal infection [73]. Although the interaction of macrophages with *C. glabrata* cells has not been thoroughly investigated so far, the authors of the study speculated that *C. glabrata*, similar to *C. albicans*, alters glucose homeostasis by inducing the glycolysis pathway, and yeasts may use the resulting glucose-depleted environment to induce macrophage death [33,67,73,74]. Furthermore, it has been shown that *C. glabrata*, after macrophage phagocytosis, inhibits the glycolysis pathway, and induces gluconeogenesis and the glyoxylate cycle [33,74]. Combining the conclusions of these reports may provide a complementary explanation for the lethality of the *Cgyps1*-*11*Δ mutant after its internalization by the host cells [36]. As studies using the *C. albicans sap6*Δ strain have also shown, the lack of Sap6 can alter the integrity of the cell wall, as well as the resistance of the strain confronted by phagocytosis by the THP-1 human monocytes [75].

## 4. Fungal Proteases as Allergens

For numerous fungal proteases, immunogenicity, defined as the ability to induce a humoral and cellular immune response, has been repeatedly indicated. Additionally, antigenicity, defined as the ability to specifically associate with the resultant effector factors of the immune response, including secreted antibodies, the major histocompatibility complex (MHC), and surface receptors present on T lymphocytes, has also been attributed to these enzymes. Furthermore, some fungal proteases are also interrelated with allergenicity, understood as the capability to stimulate a non-standard immune response, resulting in altered an physiology of the organism, the emergence of tissue destruction symptoms, and other reactions that are harmful and bothersome for the allergy-stricken individual [76].

Representatives of the *Aspergillus*, *Penicillium*, *Cladosporium*, and *Alternaria* genera are the fungi that are most frequently identified as being involved in the development of inhalant allergy and asthma in humans [77,78,79]. The initial contact of an individual with a potentially allergenic substance of fungal origin is related to sensitization–interaction, and processing by dendritic cells and antigen presentation via the MHC Class II complex, followed by the induction of T helper 2 (Th2) lymphocytes in the presence of early interleukin 4 (IL-4), and subsequent immunoglobulin E (IgE) production by B lymphocytes [80,81]. An allergic reaction, as a complex response of the immune system to re-contact with an allergen after sensitization, is associated with the binding of the foreign molecule with specific IgE on the surfaces of mast cells, followed by the release of various bioactive substances and inflammatory mediators, including histamines, leukotrienes, prostaglandins, cytokines, and serine proteases, which are all involved in the manifestation of allergy symptoms such as vasodilation, increased vascular permeability, enhanced mucus production, and the contraction of bronchial smooth muscle. Furthermore, mast cell degranulation also leads to the further activation of other immune cells, and subsequently affects epithelial cells, fibroblasts, and smooth muscle cells [80,81,82]. A serious consequence of chronic airway inflammation related to an allergic reaction may be the development of asthma—a complex disease that currently affects millions of people around the world—related to the narrowing and remodeling of the airways, and resulting in problems with breathing [83,84,85].

During the development of inhalant allergy, contact of the allergen with DC in the airway lumen, followed by the migration of immune cells through the epithelium for antigen presentation to naïve T lymphocytes, is not the only possible sensitization route for allergens that are proteases, as their enzymatic activities could greatly facilitate the direct penetration of the antigen through the epithelial layer of the respiratory tract, followed by its contact with submucosal DC [50,81,86,87,88]. Different proteases produced by fungi may alter the airway epithelium, demonstrating also the proinflammatory effect with the accompanying activation of epithelial cells, possibly through a mechanism mediated primarily by the PAR2 receptor, and the consequent calcium signaling and production of cytokines, including IL-6 and IL-8 [89,90,91,92]. The World Health Organization and International Union of Immunological Societies (WHO/IUIS) Allergen Nomenclature Database lists numerous well-documented examples of fungal proteases that are recognized as airborne allergens [93,94]. These proteases often belong to the pan-fungal allergen group, taking into account the observed IgE cross-reactivity [90]. Although most of them are alkaline and vacuolar serine proteases, some allergens also derive from the family of metalloproteases, (i.e., Asp f 5 from *A. fumigatus*), or aspartate proteases (i.e., Asp f 10 of the same species or Rhi o 1 from *Rhizopus oryzae*); additionally, even if some proteases recognized as allergens are intracellular proteins by origin, they are also repeatedly identified as being secreted extracellularly or present on the surfaces of fungal cells, and their allergenic properties are generally related to the proteolytic activity [77,95,96,97].

An interesting example of the potential of fungal proteases as the allergens is the activity of alkaline serine protease Alp1 (Asp f 13) secreted from swollen conidia of *A. fumigatus*. Alp1 is not only an important fungal virulence factor, but it is also a well-known trigger for airway hyperresponsiveness, although the complex mechanisms of its action are still being investigated [96,98]. Alp1 may induce pathophysiological RhoA-dependent Ca^2+^ sensitivity and bronchoconstriction after penetration of the bronchial submucosa and the degradation of the extracellular matrix proteins, i.e., collagens and fibronectin, leading to the destruction of their interactions with airway smooth muscle cells [99]. Moreover, Alp1 has the ability to disrupt the integrity of bronchial epithelium cells via the degradation of the junctional protein E-cadherin, followed by the activation of the mechanosensitive calcium channel TRPV4, calcium flux signaled through calcineurin, and subsequently, airway inflammation and allergic sensitization [100]. Nevertheless, the interactions of Alp1 with PAR2 or other PARs during the responses of epithelial cells remain vague [101]. Furthermore, alkaline serine protease Pen c 13 produced by *Penicillium citrinum* demonstrated the capability to activate PAR1 and PAR2 receptors on epithelial cells, increasing IL-8 production [102]. Furthermore, Pen c 13 also caused a direct disruption of junctional proteins between epithelial cells, followed by the increased penetration and production of Th2 cytokines IL-4, interleukin 5 (IL-5), and interleukin 13 (IL-13) [87]. *Penicillium chrysogenum* alkaline serine protease Pen ch 13 was demonstrated to stimulate the release of histamine by basophils, and to induce, in a protease-dependent manner, the expression of prostaglandin E2, IL-8, transforming growth factor TGF-β1, and cyclooxygenase COX-2 in epithelial cells [103,104]. Pen ch 13 also directly degraded the tight junction protein occludin, contributing to the destruction of the pulmonary epithelial barrier [104]. In the case of *Alternaria alternata*, serine proteases have been shown to be at least partially involved in the development of allergic asthma [91]; however, for this fungus, both the PAR2-dependent mechanism and the PAR2-independent route based on epidermal growth factor receptor (EGFR) activation were recently shown to be responsible for the immune response and cytokine production by epithelial cells; albeit, all fungal factors involved in this process still need to be characterized in detail [105,106,107]. In the case of Rhi o 1 aspartic endopeptidase produced by *R. oryzae*, the ability to bind IgE and to stimulate histamine release from peripheral blood mononuclear cells sensitized earlier with serum with anti-Rhi o 1 IgE antibodies was also demonstrated. This observation allowed for the classification of Rhi o 1 as an inhalant allergen [108].

Proteases from other fungi, including dermatophytes and some endemic species, also demonstrate the ability to induce an immune response, although only a few are currently confirmed allergens. They can, however, be a useful diagnostic tool, or they can also be used to prevent fungal infection by vaccination. Subtilisins and fungalysins produced by dermatophyte species possess antigenic properties and are capable of triggering immune reactions [109]. In the case of *Trichophyton* dermatophytes, proteases Sub6 and DppV may elicit an IgE-mediated immediate-hypersensitivity or delayed-type hypersensitivity responses, acting as contact allergens, as they bind IgG and IgE antibodies and stimulate cytokine production [110]. For *Microsporum canis* Sub3 subtilisin, a cell-mediated immune response was demonstrated in the experimentally infected guinea pig model, and for the extracellular metalloproteinase Mep3 of the same species, an antibody response was observed, although neither protease was protective in the vaccination trials [111,112,113,114]. In addition, the secretory aspartyl protease Mfsap1 from an opportunistic cutaneous pathogen *Malassezia furfur* has been recently indicated as strongly contributing to the development of inflammation in barrier-compromised murine skin, and the mechanisms may involve a direct interaction of protease with the host, or the Mfsap1-dependent modification of fungal cell surface hydrophobicity [115]. An endemic fungus *Paracoccidioides brasiliensis* has also been reported to produce serine and cysteine proteases that trigger IL-6 and IL-8 secretion by lung epithelial cells in PAR1- and PAR2-dependent manners [116], and representatives of the former group of *Paracoccidioides* proteases have also been demonstrated to have antigenic properties [117]. Of other endemic fungi, *Histoplasma capsulatum* produces an N-acetylated α-linked acidic dipeptidase (NAALADase) known as the most important antigen during histoplasmosis, which is advantageous for the diagnosis of this serious disease affecting mainly the lungs [118], while *Sporothrix schenckii* secretes serine and cysteine proteinases that are capable of interacting with and cleaving immunoglobulin G (IgG) [119]. The Kexin-like serine protease Kex1 produced by *Pneumocystis jirovecii*, the worldwide causative agent of pneumonia in immunocompromised individuals, has been indicated as a promising target for immunization to prevent this disease [120], and as a convenient diagnostic marker [121]. It was found that a higher risk of chronic *Pneumocystis* infections and the intensification of asthma symptoms in children with severe asthma correlated with the lowered level of protective anti-Kex1 antibodies; therefore, plasma Kex1 IgG concentration could be a supporting predictive factor for pediatric patients [122].

In general, for *C. albicans*, proteases appear to play only a minor role in Th2 and Th17 cell-driven allergic airway disease [123]. However, some Saps show immunogenic properties: for instance, the intravaginal immunization of rats with recombinant *C. albicans* Sap2 resulted in the local production of anti-Sap2 IgG and immunoglobulin A (IgA), and provided a protective effect against candidial infection by stimulating the humoral and cell-mediated immune responses against *C. albicans* infection [124,125]. Interestingly, mice immunization with *C. parapsilosis* Sap2 demonstrated enhanced survival during systemic *C. tropicalis* infection through a mixed cellular and humoral response resulting in the enhanced killing of fungal cells by neutrophils and the inhibition of biofilm formation, indicating that the protective anti-Sap2 antibodies cross-reacted with different *Candida* species [126].

## 5. Multiple Roles in Cellular Homeostasis

An effective adaptation to changing conditions in the different niches of the host organism, where changes in pH, oxidative stress, or nutrient deficiency may occur, is a prerequisite for the survival of pathogenic microorganisms. The canonical functions of extracellular proteases, based on their proteolytic activity, play a key role in the proper growth and development of fungi [4]. Interestingly, the additional moonlighting functions of these proteases have also been reported to maintain proper cellular homeostasis [66,127,128,129].

In the case of *C. glabrata*, an approach based on the use of mutants with the deletion of single or multiple *CgYPS*-encoding genes, demonstrated that these proteinases are additionally involved in thermal stress survival, the regulation of pH homeostasis, and vacuole homeostasis [66,127,128]. Under heat-induced conditions, nine *YPS* genes were overexpressed, namely *CgYPS1*, *CgYPS2*, *CgYPS4*, and *CgYPS6-11*; however, only *CgYPS1* was shown to be regulated primarily by the calcineurin-Crz1 pathway, and partially by the Slt2 MAPK pathway [127]. The *Cgyps1*Δ mutant cultured at elevated temperatures demonstrated a drastic reduction in growth, with the observed effect being restored, both after the complementation of the *YPS1* gene, and in the presence of sorbitol as an osmotic stabilizer. Together, these observations suggest that among the *C. glabrata* yapsins, predominantly Yps1 is involved in the maintenance of the cell wall integrity, preventing yeast cell lysis under heat stress [127]. Additionally, studies by Bairwa et al. demonstrated a unique role for CgYps1 in yeast survival under conditions of low external pH [66]. In the *Cgyps1Δ* mutant, intracellular acidification was noted, which consequently interfered with the regular course of physiological processes, and was partly caused by the disruption of membrane proton pump activity. In addition, the *Cgyps1*Δ mutant exhibited an increased production of ROS [66]. Although the acidic environment also increased the expression of six additional genes encoding yapsins, that is, *CgYPS2*, *CgYPS4*, *CgYPS6*, *CgYPS8*, *CgYPS10*, and *CgYPS11*, their exact functionality in regulating pH homeostasis has not been described so far [66]. As well, the role of *CgYPS* in the regulation of vacuole pH homeostasis has been revealed, and studies using the *CgYPS*-deficient mutant showed the pleiotropic effects of proteases deficiency, including the disruption of the size and internal pH of the vacuole, as well as a decrease in the activity of vacuolar membrane V-ATPase and vacuolar carboxypeptidase Y [128].

The pepsin-like aspartyl peptidase May1 (major aspartyl peptidase 1) produced by *C. neoformans* was identified as the major endopeptidase secreted under the conditions of low pH, and its activity was found to be important for fungal tolerance to acidic environments; thus, it was supposed to play a role in the *C. neoformans* ability to reside inside the phagolysosomes of macrophages [129,130]. Moreover, in the case of the *C. neoformans* deletion strain deprived of serine endopeptidase Prb1, a hypomelanization phenotype was observed, indicating the potential role of this protease in the process of melanization, which is crucial for the virulence of this fungus [129].

An interesting example of a protease that is a moonlighting protein is the serine endopeptidase PnmB produced by *Aspergillus nidulans*. This fungal species is characterized by a low pathogenicity, and it can cause serious diseases only in people with primary immunodeficiency disorder—chronic granulomatous disease [131]. PnmB plays an important role in nutrient acquisition and nitrogen metabolism. It may be equipped with the signal peptide directing protease to the outside of the cell, where it degrades available proteins, thus providing a nitrogen source for the fungus. In addition, PnmB may exhibit a more sophisticated, regulatory role intracellularly, where the truncated form of the protein functions. Under conditions of nitrogen starvation, PnmB is involved in controlling the activity of GATA transcription factor AreA through the proteolytic degradation of its co-repressor NmrA, leading to the subsequent activation of genes that are involved in nutrient acquisition and metabolism, and in a positive regulatory feedback mechanism, enhancing *PnmB* transcription by AreA [132]. Interestingly, the extracellular and intracellular forms of PnmB differ not only in the presence of a signal peptide for secretion, but also in substrate specificity, with a broad spectrum of activity being exhibited by the secreted form, and a high specificity that is limited to NmrA, but the underlying mechanisms have not been fully recognized [132,133].

The problem of resistance to antimicrobial drugs is particularly important now in the case of fungal infections. Several reports have shown an association between a decreased susceptibility to antifungal drugs and an increased degree of Sap production by *Candida* species [134]. Resistance to antifungal drugs was correlated with biofilm formation and the secretion of Saps, particularly Sap2, Sap9, and Sap10; however, the exact mechanisms are still unknown [18,135,136].

## 6. Conclusions

Since the very beginning of research on the virulence factors of human pathogenic fungi, an important role has been attributed to fungal proteases, emphasizing the role of their proteolytic activity in the acquisition of nutrients and the degradation of host protective barriers during the development of infection. So far, many interesting examples have been documented that relate the mechanisms of fungal pathogenesis to the proteolytic degradation of host bioactive peptides, and structural and regulatory proteins. However, in addition to the canonical function of these fungal enzymes, functionalities that are independent of proteolytic activity have also been indicated for selected representatives of fungal proteases, some of which are related to the impairment of host defense mechanisms and the modulation of immune response, as well as the induction of an exaggerated immune reaction, followed by the development of an allergy. These additional roles often reveal atypical protease functions that are often independent of hydrolytic degradation, and thus, they may place the protease in the group of moonlighting proteins. Moreover, once released into the external environment, proteases can act at remote sites in the host organism. Therefore, it may complicate insight into the participation of proteases in the pathogenesis of fungal infection, and hinder the inhibition of some processes during antifungal treatment, based on the elimination of proteolytic activity. Therefore, knowledge of the multitasking proteases is essential for understanding and for adequately controlling fungal diseases.

## Figures and Tables

**Table 1:** Examples of extracellular proteases produced by selected fungi that are pathogenic for humans.

Fungal Species	Identified Proteases	Protease Classification
*Candida* spp.		
*C. albicans*	Sap1-Sap10	aspartic proteases
*C. glabrata*	yapsins (CgYps1-11)	aspartic proteases
*C. parapsilosis*	Sapp1, Sapp2	aspartic proteases
*Aspergillus* spp.		
	secreted subtilisins (i.e., Alp1/Asp f 13)	serine proteases
	elastinolytic proteases Mep, fungalysins (i.e., Asp f 5)	metalloproteases
	secreted aspartyl endopeptidase (i.e., aspergillopepsin-1/ Asp f 10)	aspartic proteases
	protease PnmB	serine protease
*Penicillium* spp.		
	secreted subtilisin-like proteases (i.e., Pen c 13, Pen ch 13)	serine proteases
*Cryptococcus neoformans*		
	major aspartyl peptidase 1 May1	aspartic protease
	endopeptidase Prb1	serine protease
	secreted metalloprotease Mpr1	metalloprotease
*Pneumocystis jiroveci*	kexin (Kex1)	serine protease
*Rhizopus oryzae*	rhizopuspepsin, Rhi o 1	aspartic protease
*Malassezia furfur*	secretory aspartyl protease Mfsap1	aspartic protease
Dermatophytes		
	secreted subtilisins (Sub2–Sub7)	serine proteases
	fungalysins (Mep1, Mep3, Mep4)	metalloproteases
	dipeptidyl peptidases (DppIV, DppV)	serine exoproteases
Endemic fungi		
*Paracoccidioides brasiliensis*	extracellular subtilisin-like protease (*Pb*SP)	serine protease
*Histoplasma capsulatum*	N-acetylated α-linked acidic dipeptidase (NAALADase)	metallocarboxypeptidase
*Sporothrix schenckii*	proteinase I	serine protease
	proteinase II	cysteine protease

## Data Availability

No new data were created or analyzed in this study. Data sharing is not applicable to this article.
